# Surgical Myectomy with Anterior Mitral Leaflet Extension Versus Isolated Myectomy in Patients with Hypertrophic Obstructive Cardiomyopathy

**DOI:** 10.1055/a-2768-2815

**Published:** 2026-01-07

**Authors:** Tijn J.P. Heeringa, Marieke Hoogewerf, Romy Hegeman, Dimitri van Wylick, David Stecher, Maarten Jan Cramer, Giulia De Zan, Yvonne Koop, Ronald C.A. Meijer, Nicolaas P.A. Zuithoff, Pim van der Harst, Marco Guglielmo, Ilonca Vaartjes, Mostafa M. Mokhles, Niels P. van der Kaaij

**Affiliations:** 1 Department of Cardiothoracic Surgery, University Medical Centre Utrecht, Utrecht, The Netherlands; 2 Department of Cardiothoracic Surgery, St Antonius Hospital, Nieuwegein, Utrecht, The Netherlands; 3 Department of Cardiothoracic Surgery, Amsterdam University Medical Centres, Amsterdam, The Netherlands; 4 Department of Cardiothoracic Surgery, Medical Centre Leeuwarden, Leeuwarden, The Netherlands; 5 Department of Cardiology, University Medical Centre Utrecht, Utrecht, The Netherlands; 6 Department of Cardiovascular Epidemiology, Julius Center for Health Sciences and Primary Care, Utrecht, The Netherlands

**Keywords:** cardiomyopathy, mitral valve surgery, surgery, complications

## Abstract

**Background:**

This study evaluated the echocardiographic parameters and complication rates of surgical myectomy with concomitant anterior mitral leaflet extension (SM + AMLE) and isolated SM in hypertrophic obstructive cardiomyopathy (HOCM) patients.

**Methods:**

All HOCM patients undergoing SM + AMLE (2006–2015) and isolated SM (2015–2020) in our centre were analysed. The primary outcome was left ventricular outflow tract (LVOT)-gradient and surgical reoperation (SM/mitral surgery). Secondary outcomes were aortic cross-clamping (ACC) time, iatrogenic ventricular septal defect (VSD), and mortality at 30-day and 3-year follow-up. Mixed-effects models assessed postoperative changes in LVOT-gradient measurements over time until a 3-year follow-up.

**Results:**

This cohort ( *n* = 59) consisted of 34 (58%) SM + AMLE and 25 (42%) isolated SM procedures. There were 32 (54%) males and 27 (46%) females with a mean age of 55 ± 13 years at the time of the intervention. Postoperatively, no differences were observed over time in the median LVOT-gradient ( *p* = 0.34). In the SM + AMLE group, 6% ( *n* = 2) required surgical reoperation (due to patch dehiscence) versus 0% in the SM group. In the SM + AMLE group, the ACC time was significantly higher (86 minutes [interquartile range [IQR]: 74–103]) than in the isolated SM group (48 minutes [IQR: 39–57]; *p* < 001). In both groups, the VSD complication rate was 0%, and neither procedure led to death at 3-year follow-up.

**Conclusion:**

HOCM-patients who underwent SM + AMLE had comparable clinical and echocardiographic outcomes to patients who underwent isolated SM. This suggests that increasing procedural complexity may not improve outcomes. However, given potential confounding, this should be interpreted with caution, future prospective randomised controlled trials are necessary.

## Introduction

Hypertrophic cardiomyopathy (HCM) is the most prevalent inherited cardiomyopathy, affecting about 1 in every 500 individuals of the general adult population. 1 Hypertrophic obstructive cardiomyopathy (HOCM) is characterised by a dynamic left ventricular outflow tract (LVOT)-obstruction, which is caused by: (1) a thickened interventricular septum, (2) systolic anterior motion (SAM), (3) an anatomically narrowed LVOT, and (4) possible structural abnormalities of the mitral valve apparatus and papillary muscle orientation. 2 The mitral valve can play a significant role in the obstruction, whereby SAM further accentuates hemodynamics with secondary mitral regurgitation, and the LVOT-obstruction, during the essential and already-compromised systolic phase.

According to the 2023 European Society of Cardiology Guidelines for the management of cardiomyopathies, surgical myectomy may performed in combination with mitral valve repair or replacement to further reduce LVOT-obstruction and surgical reoperation rates. 1 3 Specifically, mitral valve surgery should be considered in symptomatic patients with a resting or provoked LVOT gradient ≥ 50 mm Hg and moderate-to-severe mitral regurgitation that cannot be corrected by surgical myectomy alone (Class IIa, Level of Evidence C).

However, in almost all cases, isolated surgical myectomy significantly reduces the LVOT-gradient, mitral regurgitation, and SAM, thus directly reducing symptoms and improving the quality of life. 4 5 Thereby, the addition of mitral valve surgery leads to longer cardiopulmonary bypass (CPB) and aortic cross-clamp (ACC) times as well as additional tissue trauma. However, a large cohort study encompassing 1,248 isolated surgical myectomy procedures and 799 surgical myectomy in combination with mitral valve surgery demonstrated no discernible contrast in survival rates that emerged over a follow-up period of 6.2 ± 4 years. 6 Although surgical myectomy with concomitant mitral valve surgery is safe, its further benefit in the context of surgical myectomy has not been proved. 7 Additional details regarding echocardiographic parameters and variations in complication rates are lacking between these groups.

It is clear that evidence regarding detailed clinical and echocardiographic outcomes of the addition of mitral valve surgery to surgical myectomy versus isolated surgical myectomy is still lacking and remains a topic of controversy. In this study, the authors evaluate the clinical and echocardiographic outcomes after surgical myectomy with anterior mitral leaflet extension (SM + AMLE) and isolated surgical myectomy in HOCM patients.

## Patients and Methods

### Design, Setting, and Study Population

All patients, aged ≥ 18 years who underwent a surgical myectomy at the University Medical Centre (UMC) Utrecht between January 1, 2006, and December 31, 2020, were included in this study. Patients were diagnosed with HCM and underwent diagnostic verification by two experienced imaging cardiologists (M.G. and M.J.C.) with more than 10 years of experience in performing and reporting echocardiography, using electronic health records and echocardiography in accordance with the American College of Cardiology/American Heart Association 2020 guidelines. HCM was diagnosed if myocardial end-diastolic wall thickness was ≥15 mm in the absence of any other disease responsible for hypertrophy. 8 The local institutional ethical review board approved this study (22/075; February 2, 2022), and all patients provided written informed consent. Data collection and follow-up are described in the Supplementary Data S1.1 (available in the online version only). The methods used for parameter evaluation can be found in the Supplementary Data S1.2 (available in the online version only).

### Surgical Indication

The indication for surgical myectomy was determined preoperatively, in patients with HOCM who showed a resting or provoked LVOT gradient exceeding 50 mm Hg, despite optimal medical therapy. This indication remained unchanged throughout the study period. The indication to change isolated surgical myectomy for SM + AMLE was determined intraoperatively, when the surgeon considered isolated surgical myectomy unlikely to provide an optimal result. Factors favouring the SM + AMLE technique included larger anterior mitral valve, atrial fibrillation, limited septal hypertrophy, pronounced SAM of the mitral valve, and significant mitral regurgitation. 9 In the authors' centre, SM + AMLE was the preferred surgical procedure prior to 2016 and no isolated surgical myectomy procedures were performed in that period of time. Thereafter, the isolated surgical myectomy procedure became the preferred procedure due to a change in surgeons, as the new surgeon introduced a different perspective on the procedure. After 2016, SM + AMLE was no longer performed, while the patient selection criteria for surgical myectomy remained unchanged. Transition provided an opportunity to evaluate two distinct patient groups within the same centre: (1) HOCM patients who underwent SM + AMLE and (2) HOCM patients who underwent isolated surgical myectomy.

All patients were discussed in structured dedicated heart team meetings including imaging cardiologists and cardiac surgeons. The included patients were operated on by three consecutive surgeons. The SM + AMLE group was operated on by three surgeons in the period from January 2006 to January 2016, with one surgeon performing the majority of the procedures (78%) during this period. All isolated surgical myectomy procedures were performed by a single surgeon in the period from January 2016 to December 2020. All patients underwent a surgical myectomy as developed by Morrow et al. 10 In the SM + AMLE group, the AMLE was performed after the surgical myectomy was completed, using the same transverse aortotomy for access. The AMLE procedure has been described previously. 9

### Outcomes

The primary outcome measures included postoperative LVOT-gradient and surgical reoperation. The LVOT-gradient was measured as a continuous variable at four time points: (1) preoperatively; (2) postoperatively during hospital admission; (3) at first outpatient follow-up, four4 to 52 weeks after surgery; and (4) at a 3-year (range: 2–3.5 years) follow-up after surgery. Surgical reoperation was defined as a second surgical procedure (surgical myectomy, mitral valve repair, and mitral valve replacement) during hospital admission or within 30 days and at 3-year follow-up. The secondary outcome measures were ACC time, iatrogenic ventricular septal defect (VSD) repair, early mortality (either 30-day or during hospital admission), and mortality at 3-year follow-up. Mortality was defined as all-cause mortality. The complications were defined as early complications (either 30-day or during hospital admission) and at 3-year follow-up. The following echocardiogram measurements—interventricular septal thickness in diastole (IVSd), left atrial volume index (LAVI), left atrial dimension index (LADI), tricuspid annular plane systolic excursion (TAPSE), and left ventricular ejection fraction (LVEF)—were also collected at the four time points.

### Statistical Analysis

Normally distributed continuous variables were presented as mean ± standard deviation and compared using the Student's *t* -test. Skewed continuous variables were presented as median and interquartile range (IQR) and compared using the Mann–Whitney U test. The normality of the distributions was tested by visual inspection of the Q–Q (quantile–quantile) plot and the Kolmogorov–Smirnov test. Categorical variables were presented as counts and percentages and compared using the χ2 test or Fisher's exact test, as appropriate. Clopper–Pearson exact method was used to calculate the confidence intervals (CIs) for proportions.

Sensitivity analyses were performed for patients with and without SAM preoperatively and were performed for excluded patients stratified for concomitant procedures. The stratified concomitant procedures can be found in the Supplementary Table S1 (available in the online version only).

Echocardiographic measurements were analysed over time with linear mixed-effects models. Mixed-effects modelling allows for more accurate analysis of dependent data, such as measurements collected over time on the same individuals. 11 This approach of longitudinal data analysis is also proposed by the 2008 guideline for reporting mortality and morbidity after cardiac valvular interventions. 12 The model structure is described in detail in the Supplemental Data S2 (available in the online version only).

Missing data were observed for some variables; details are presented per variable in the Supplementary Tables S2 and S3 (available in the online version only).

Data were analysed with the SPSS for macOS version 29.02.0 (SPSS, Inc., Chicago, Illinois, United States). The statistics program R (the R Foundation for Statistical Computing, Vienna, Austria, Version 4.1.2). All statistical tests with a *p* -value ≤ 0.05 were considered significant.

## Results

### Patient Characteristics

**Fig. 1:**
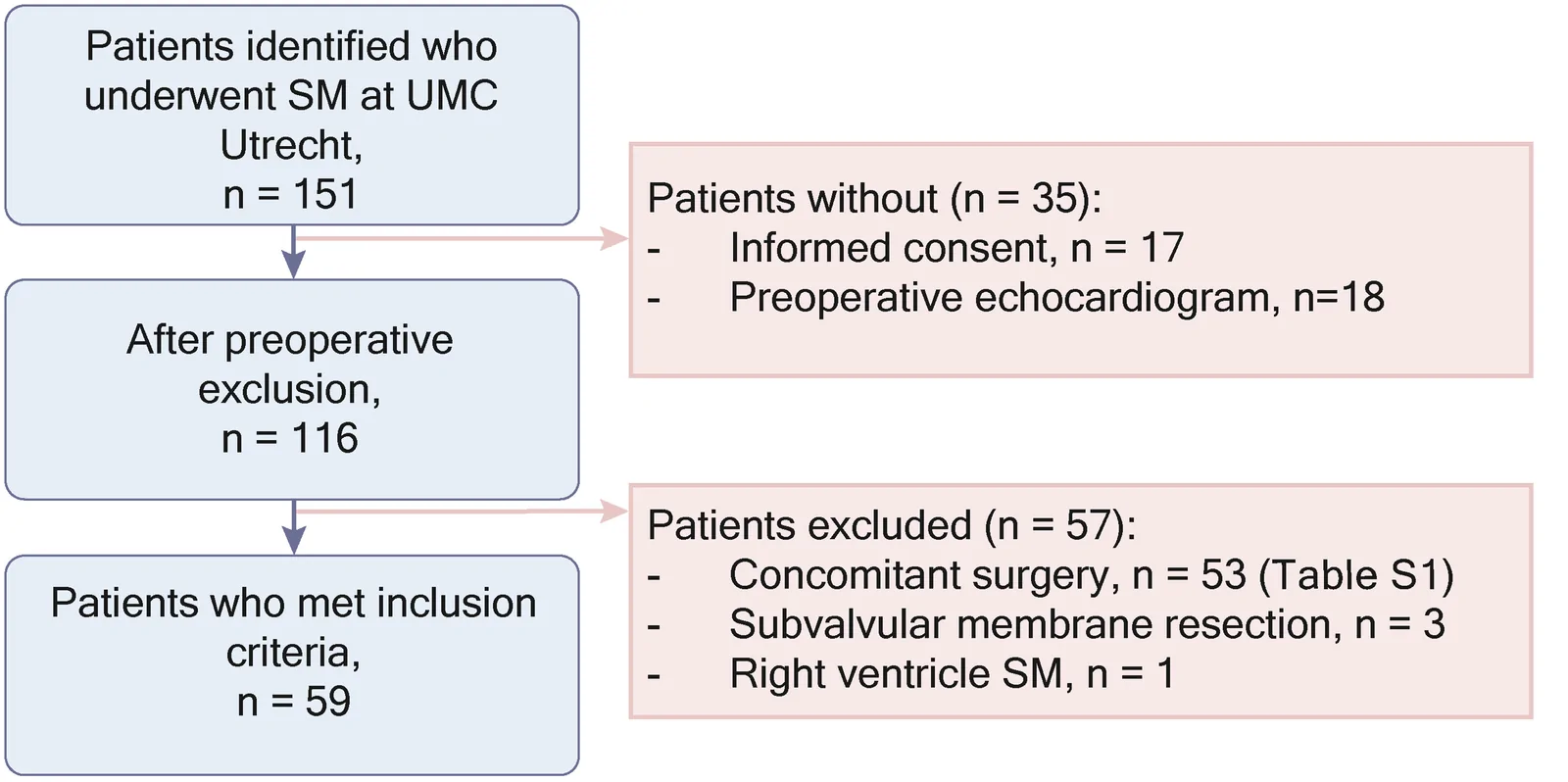
Flowchart demonstrating a selection of hypertrophic obstructive cardiomyopathy patients operated with a surgical myectomy procedure.

Between January 2006 and December 2020, a total of 151 patients underwent a surgical myectomy at the UMC Utrecht. Fig. 1 depicts the flowchart of patients who were finally included in this study ( *n* = 59). In the SM + AMLE group, 34 (58%) patients were operated on between January 2006 and January 2016, and in the isolated surgical myectomy group, 25 (42%) patients were operated on between January 2016 and January 2020. In the SM + AMLE group, patients had a mean age of 53 ± 13 years compared with the isolated surgical myectomy group patients' mean age of 58 ± 12 years ( *p* = 0.09). In the SM + AMLE group, patients exhibited a significantly lower provoked LVOT-gradient (78 ± 28 mm Hg) than in the isolated surgical myectomy group (96 ± 22 mm Hg; *p* = 0.03). Table 1 displays preoperative patient characteristics for the SM + AMLE group and the isolated surgical myectomy group; percentages for missing values can be found in Supplementary Table S2 (available in the online version only).

**Table 1:** Baseline patient characteristics

Characteristics	SM + AMLE	Isolated SM	
	( *n* = 34)	( *n* = 25)	*p* -Value
Age, y, mean (SD)	53 (13)	58 (12)	0.09
Male sex, *n* (%)	19 (56)	13 (52)	0.72
BMI, kg/m ^2^ , mean (SD)	27 (5)	26 (4)	0.54
Diabetes, *n* (%)	3 (9)	2 (9)	>0.99
Creatinine, µmol/L, mean (SD)	84 (19)	77 (17)	0.12
Prior cardiac surgery, *n* (%)	2 (6)	1 (4)	>0.99
Prior ASA, *n* (%)	2 (6)	0	0.50
Atrial fibrillation history, *n* (%)	4 (12)	5 (20)	0.26
ICD history, *n* (%)	3 (9)	3 (12)	>0.99
Pacemaker history, *n* (%)	1 (3)	0	>0.99
LBBB, *n* (%)	6 (18)	3 (12)	0.72
RBBB, *n* (%)	3 (9)	0	0.25
Pathogenetic DNA variants a	12 (70)	12 (80)	>0.99
NYHA, *n* (%)			0.48
I–II	14 (41)	7 (28)	
III–IV	20 (59)	18 (72)	
Echocardiographic			
Systolic LVF, *n* (%)			0.44
Good (EF ≥ 50%)	34 (100)	24 (96)	
Impaired (EF 40–50%)	0	1 (4)	
Moderately reduced (EF 30–40%)	0	0	
LAVI, mL/m ^2^ , mean (SD)	52 (16)	47 (18)	0.14
LAVI, *n* (%)			0.21
Normal + mild	9 (26)	12 (48)	
Moderate + severe	23 (67)	13 (52)	
LADI, mm/m ^2^ , mean (SD)	22 (4)	20 (3)	0.10
Resting LVOT gradient, mm Hg, mean (SD)	50 (26)	44 (28)	0.45
Provocative LVOT gradient, mm Hg, mean (SD)	78 (28)	96 (22)	0.03
Mitral regurgitation, *n* (%)			0.45
Grade 1 + 2	23 (68)	20 (80)	
Grade 3 + 4	11 (32)	5 (20)	
Valvular SAM, *n* (%)	33 (97)	21 (84)	0.15
AML length, mm, mean (SD)	30 (4)	29 (4)	0.51
IVSd, mm, mean (SD)	23 (4)	22 (4)	0.80
TAPSE, mm, mean (SD)	21 (3)	21 (3)	0.80
Medication, *n* (%)			
ACE-inhibitors	4 (12)	4 (16)	0.72
Anticoagulation	8 (24)	6 (24)	>0.99
Amiodaron	2 (6)	0	0.50
ARB	3 (9)	3 (12)	0.69
Beta-blockers	28 (82)	22 (88)	0.72
Calcium channel blockers	7 (21)	7 (28)	>0.99
Disopyramide	2 (6)	2 (8)	>0.99
Diuretics	3 (9)	1 (4)	0.63

Abbreviations: ACE, angiotensin-converting enzyme; ARB, angiotensin II receptor blocker; ASA, alcohol septal ablation; BMI, body mass index; ICD, implantable cardioverter defibrillator; IQR, interquartile range; IVSd, interventricular septal thickness in diastole; LADI, left atrial dimension indexed; LAVI, left atrial volume index; LBBB, left bundle branch block; LVF, left ventricular function; LVOT, left ventricular outflow tract; NYHA, New York Heart Association; RBBB, right bundle branch block; SAM, systolic anterior motion; SD, standard deviation; TAPSE, tricuspid annular plane systolic excursion.

Note: Values are mean ± SD, median [IQR], or *n* (%).

a Proportion of genotype-positive patients of genetically tested group.

### Left Ventricular Outflow Tract-Gradient and Surgical Reoperation

**Fig. 2:**
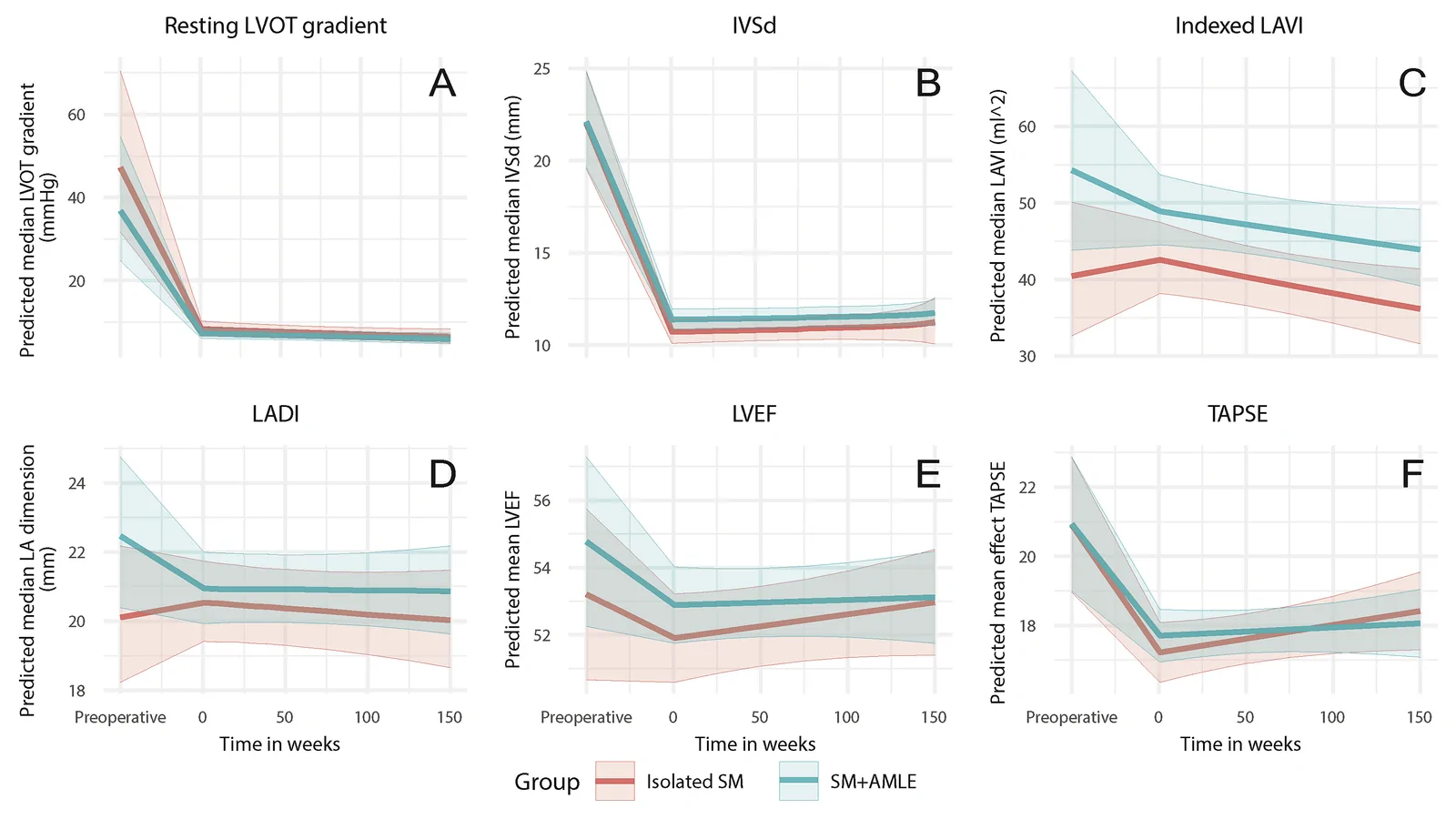
Mixed-effect models of echocardiographic variables between HOCM patients who underwent SM + AMLE and HOCM patients who underwent isolated surgical myectomy; LVOT gradients (A), IVSd (B), LAVI (C), LADI (D), LVEF (E), and TAPSE (F). HOCM, hypertrophic obstructive cardiomyopathy; IVSd, interventricular septal thickness in diastole; LADI, left atrial dimension indexed; LAVI, left atrial volume index; LVEF, left ventricular ejection fraction; LVOT, left ventricular outflow tract; SM, surgical myectomy; SM + AMLE, surgical myectomy with anterior mitral leaflet extension; TAPSE, tricuspid annular plane systolic excursion.

The postoperative resting LVOT-gradient based on the model over time was not significantly different between both groups (Wald test: *p* = 0.34). In the SM + AMLE group, the median resting LVOT-gradient declined from 36 mm Hg (95% CI: 25–55) to 7 mm Hg (95% CI: 7–8) at hospital discharge. In the isolated surgical group, the median resting LVOT-gradient based on the mixed model declined from 47 mm Hg (95% CI: 32–70) to 8 mm Hg (95% CI: 7–10) at hospital discharge. Fig. 2A depicts time-related changes in resting LVOT-gradient between both groups. Sex and age were not associated with the resting LVOT-gradient.

In the SM + AMLE group, three patients exhibited provoked LVOT-gradients exceeding 30 mm Hg (42, 35, and 37 mm Hg) during postoperative hospital admission, whereas their corresponding resting LVOT-gradients were 27, 27, and 22 mm Hg, respectively. None of these patients demonstrated SAM. Consequently, it was decided not to proceed with a surgical reoperation. In the isolated surgical myectomy group, two patients had a residual resting gradient exceeding 30 mm Hg. However, one patient opted against a surgical reoperation, and the other patient was decided not to proceed with a surgical reoperation due to a LVOT-gradient of 31 mm Hg.

At 3-year follow-up, patients in the SM + AMLE group had a surgical reoperation rate of 6% ( *n* = 2) compared with the isolated surgical myectomy group of 0%. These two patients required surgical reoperation on the mitral valve due to patch dehiscence after a follow-up period of 10 and 21 months, respectively.

### Surgery and 30-day Complication Rates

In the SM + AMLE group, patients had a prolonged ACC time (86 [74–103] vs. 48 minutes [39–57]; *p* < 0.001) and a prolonged CPB time (109 [94–136] vs. 69 minutes [59–92]; *p* < 0.001). Neither procedure led to iatrogenic VSD repair and neither procedure led to deaths. Furthermore, neither procedure led to a new implantable cardioverter defibrillator implantation, and no strokes occurred. The new permanent pacemaker rate was 9% (95% CI: 2–9) in the SM + AMLE group compared with 4% (95% CI: 1–20) in the isolated surgical myectomy group ( *p* = 0.623). In the SM + AMLE group, the incidence of heart rhythm complications (53% [95% CI: 35–70]) was significantly higher than in the isolated surgical myectomy group (48% [95% CI: 28–68]; *p* = 0.012). In the SM + AMLE group, two patients required a mitral valve replacement during a second pump run. One case (3%) was due to residual moderate mitral regurgitation caused by prolapse and one case (3%) was due to residual LVOT-gradient (100 mm Hg) caused by SAM.

### Echocardiographic Parameters

During the study period, 267 echocardiograms were reviewed for 59 subjects. Fig. 2 depicts time-related changes in IVSd ( Fig. 2B ), LAVI ( Fig. 2C ), LADI ( Fig. 2D ), LVEF ( Fig. 2E ), and TAPSE ( Fig. 2F ), between both groups. The descriptives of these echocardiographic variables are provided in the Supplementary Table S4 and Supplementary Fig. S1 (available in the online version only). In the SM + AMLE group, the median LAVI over time was significantly higher than in the isolated surgical myectomy group ( *p* = 0.01). Resting LVOT-gradient, IVSd, LADI, LVEF, and TAPSE were similar between both procedures during follow-up ( Supplementary Table S3 , available in the online version only).

### Follow-up

#### Clinical Outcomes

**Fig. 3:**
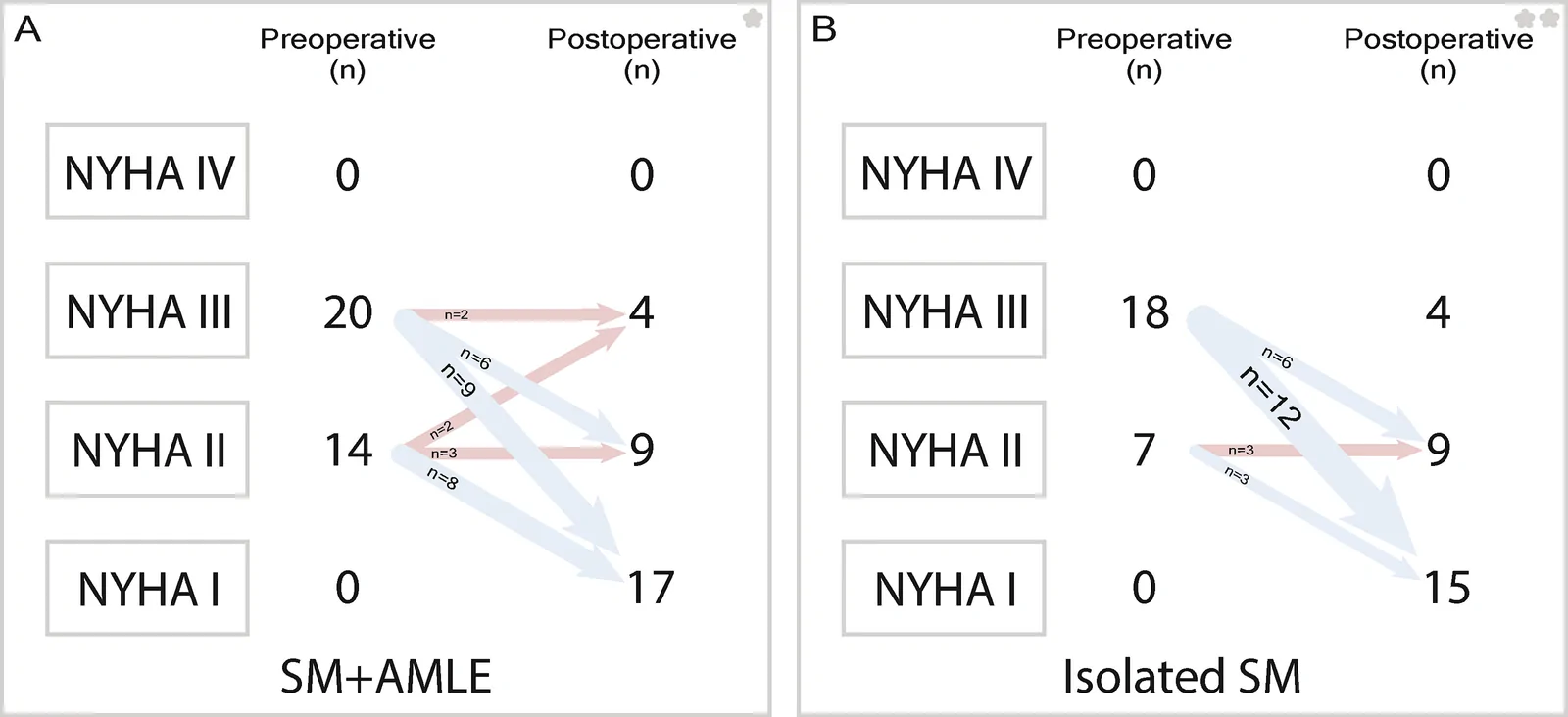
Preoperative NYHA functional class, NYHA functional class at last follow-up, and the difference in NYHA functional class; (A) HOCM patients who underwent SM + AMLE, (B) HOCM patients who underwent isolated surgical myectomy. *4 missing values, **1 missing value. NYHA, New York Heart Association.

Both groups were evaluated at the 3-year follow-up and exhibited similar complication rates ( Table 2 ). Echocardiographic findings at the 3-year follow-up have been described above. New York Heart Association (NYHA) classification outcomes are summarised in Fig. 3 and descripted in Supplementary Data S1 (available in the online version only).

**Table 2:** Intraoperative, postoperative, and follow-up clinical outcomes

Characteristics	SM + AMLE	Isolated SM	
	( *n* = 34)	( *n* = 25)	*p* -Value
Intraoperative			
VSD, *n* (%)	0	0	NA
ACC, time, min, median [IQR]	86 [74–103]	48 [39–57]	0.001
CPB, time, min, median [IQR]	109 [94–136]	69 [59–92]	0.001
MV repair to MV replacement switch, *n* (%)	2 (6)	–	NA
Rhythm surgery	2 (6)	4 (16)	0.386
Echocardiographic (first after surgical myectomy)			
Delta resting LVOT gradient max, mm Hg, median [[IQR]	58 [41–80]	79 [68–92]	0.008
Delta resting LVOT gradient, mm Hg median [IQR]	41 [20–49]	32 [9–50]	0.55
Resting LVOT gradient, mm Hg, median [IQR]	9 [5–10]	8 [5–10]	0.9
Residual LVOT gradient, *n* (%)			
Resting (≥30 mm Hg)	0	2 (8)	NA
Provocative (≥50 mm Hg)	0	1 (4)	NA
Mitral regurgitation, *n* (%)			0.57
Grade 1 + 2	33 (97)	23 (92)	
Grade 3 + 4	1 (3)	2 (8)	
Delta mitral regurgitation, *n* (%)			
Stable grade 1 + 2	22 (65)	19 (76)	0.40
Stable grade 3 + 4	0	1 (4)	NA
Progression grade 1 + 2 to grade 3 + 4	1 (3)	1 (4)	NA
Regression grade 3 + 4 to grade 1 + 2	11 (32)	4 (16)	0.23
SAM, *n* (%)	0	2 (8)	NA
Postoperative (either at 30-day or during hospital admission)			
Mortality, *n* (%)	0	0	NA
New pacemaker, *n* (%)	3 (9)	1 (4)	0.130
New ICD, *n* (%)	0	0	NA
Stroke, *n* (%)	0	0	>0.99
Heart rhythm complications, *n* (%)	18 (53)	12 (48)	0.012
LBBB, *n* (%)	32 (94)	24 (96)	NA
Surgical reoperation, *n* (%)			
Mitral valve surgery	0	0	NA
Residual obstruction	0	0	NA
Re-exploration for sternum wound problems	0	1 (4)	NA
Re-exploration for bleeding	2 (6)	2 (8)	NA
Three-year follow-up, *n* (%)			
Mortality	0	0	NA
New pacemaker	3 (9)	2 (4)	0.501
New ICD	0	0	NA
Stroke	0	0	NA
Surgical reoperation			
Mitral valve surgery a	2 (6)	0	NA
Residual obstruction ( *n* , [%])	0	0	NA
Re-exploration for sternum wound problems	0	1 (4)	NA

Abbreviations: ACC, aortic cross-clamp; AMLE, anterior mitral leaflet extension; CPB, cardiopulmonary bypass; ICD, implantable cardioverter defibrillator; IQR, interquartile range; LBBB, left bundle branch block; MV, mitral valve; NA, not applicable; SAM, systolic anterior motion; SD, standard deviation; VSD, ventricular septal defect.

Note: Values are mean ± SD, median [IQR], or *n* (%).

a Due to patch dehiscence.

#### Sensitivity Analysis

No differences were exhibited in baseline characteristics, clinical outcomes, and echocardiographic outcomes between patients with and without SAM preoperatively. In this cohort, the mean age was significantly lower than in patients who were excluded due to concomitant surgery ( *p* < 0.001).

## Discussion

According to the 2023 European Society of Cardiology Guidelines for the management of cardiomyopathies, some HOCM patients benefit from surgical myectomy and concomitant mitral valve surgery. 1 However, the clinical and echocardiographic outcomes of concomitant mitral valve surgery remain unclear. In this study, HOCM patients undergoing SM + AMLE had similar outcomes to those undergoing isolated surgical myectomy, including comparable echocardiographic LVOT-gradient measurements during follow-up. These findings support the notion that SM + AMLE does not necessarily provide additional clinical or echocardiographic benefits over isolated surgical myectomy.

In this study, both procedures effectively relieved LVOT-obstruction with comparable complication rates, consistent with findings from high-volume centres. 3 6 13 14 15 For instance, the intraoperative VSD complication rate was 0% for both procedures. 3 Additionally, neither procedure resulted in deaths at the 3-year follow-up, aligning with outcomes reported in previous large studies. 6 16 17

However, the procedural outcomes did differ. Patients who underwent SM + AMLE had significantly longer ACC and CPB times compared with those undergoing isolated surgical myectomy. Additionally, although not statistically significant, the 30-day permanent pacemaker implantation rates were higher in the SM + AMLE group. 18 Similarly, the surgical reoperation rate at 3-year follow-up was higher in SM + AMLE patients, primarily due to patch dehiscence, which aligns with previous research on SM + AMLE. 7 19

The role of mitral valve surgery in HOCM patients without intrinsic mitral valve disease remains controversial, largely due to methodological limitations in the current literature and an incomplete understanding of the underlying pathophysiological mechanisms. 13 16 17 In the Netherlands, the SM + AMLE procedure was introduced in 1986 and has been performed at several Dutch academic centres. 9 Initially, the SM + AMLE procedure was preferred over mitral valve replacement in HOCM patients, because it was better able to prevent thromboembolic complications, persistent residual LVOT-obstruction, and residual SAM-related mitral regurgitation. Today, at some Dutch academic centres, SM + AMLE is indicated only in HOCM patients with enlarged anterior mitral leaflets (>12 cm ^2^ ). 20 Although the SM + AMLE procedure is safe, the benefit of concomitant AMLE in the context of surgical myectomy has not been proved. 7 The findings of the present study support this trend, indicating that SM + AMLE adds procedural complexity, increases operative time, and may introduce additional potential complications

### Strengths and Limitations

This study has several strengths. Both procedures were performed for similar indications, with mitral valve surgery primarily aimed at preventing residual LVOT-obstruction and SAM-related mitral regurgitation, rather than treating intrinsic mitral valve disease. Baseline characteristics were comparable between groups. Moreover, the study includes detailed clinical and longitudinal echocardiographic data, enabling robust data analysis over time. This allowed for valuable insights into echocardiographic measurements over time in both groups. Furthermore, all surgical myectomy procedures were performed at a relatively high-volume hospital by three surgeons with specific expertise in the surgical myectomy procedure. Although two surgeons were trained by the first, surgeon-specific variability in patient outcomes, beyond differences in surgical technique, could not be excluded.

However, this study has several limitations. First, the inclusion periods differed between groups, potentially introducing performance bias and preventing comparison of the NYHA functional class at the most recent follow-up. Second, the mitral valve area was not measured, despite previous studies advocating SM + AMLE in patients with an enlarged anterior mitral leaflet (>12 cm ^2^ ). To address this, we ensured similar procedural indications and performed a sensitivity analysis for patients with preoperative SAM. Previous studies have demonstrated that anterior mitral leaflet length is not a factor of LVOT-obstruction after surgical myectomy. 21 Still, a subgroup analysis of patients with and without an enlarged anterior mitral leaflet could have provided further clarity on when SM + AMLE might be beneficial. Third, postoperative provoked LVOT-gradient measurements were less complete (33%), which may have led to missed or underestimated latent obstruction, potentially affecting the interpretation of the results. Fourth, not all echocardiograms were performed using the same equipment, which may have contributed to variations in echo quality. However, all transthoracic echocardiography (TTE) were reassessed by experienced imaging cardiologists. Finally, the low incidence of complications limited the ability to statistically analyse secondary clinical outcomes. We acknowledge that the available evidence remains limited to support definitive conclusions. However, the role of mitral valve repair in patients without intrinsic mitral valve disease remains controversial. Accordingly, publication of all available data is essential to facility future meta-analyses.

## Conclusion

This study demonstrated similar clinical and echocardiographic outcomes in HOCM patients undergoing SM + AMLE and isolated surgical myectomy; although the SM + AMLE group had a surgical reoperation rate of 6% compared with the isolated surgical myectomy group of 0%, this difference did not reach statistical significance. These findings support the notion that increasing procedural complexity does not lead to improved outcomes. However, potential confounding factors cannot be excluded; thus, these results should be considered hypotheses-generating and may warrant further evaluation in future prospective randomised controlled trials. Although such trials are highly unlikely given the heterogeneity of the patient population and the limited number of eligible patients following the introduction of myosin inhibitor therapy. While some HOCM patients may benefit from SM + AMLE, further research is required to identify the specific patient characteristics that would justify its use.

## Clinical Practice Points

- This study had similar longitudinal echocardiographic parameters and 30-day and 3-year follow-up complication rates between HOCM patients without intrinsic mitral valve disease undergoing SM + AMLE and isolated surgical myectomy.
- SM + AMLE increases procedural complexity, including potential complications as well as being time-consuming, while it does not lead to improved outcomes.
- In the authors' hospital, the incidences of complication rates are in line with the complication rates reported by high-volume centres.
- In the authors' hospital, isolated surgical myectomy will continue to be performed in HOCM patients without intrinsic or degenerative mitral valve disease.

## References

[1] ESC Scientific Document Group Arbelo E Protonotarios A Gimeno J R 2023 ESC Guidelines for the management of cardiomyopathies Eur Heart J 2023 44 37 3503 3626 37622657 10.1093/eurheartj/ehad194

[2] Guigui S A Torres C Escolar E Mihos C G Systolic anterior motion of the mitral valve in hypertrophic cardiomyopathy: a narrative review J Thorac Dis 2022 14 06 2309 2325 35813751 10.21037/jtd-22-182 PMC9264047

[3] Kotkar K D Said S M Dearani J A Schaff H V Hypertrophic obstructive cardiomyopathy: the Mayo Clinic experience Ann Cardiothorac Surg 2017 6 04 329 336 28944173 10.21037/acs.2017.07.03 PMC5602208

[4] Nguyen A Schaff H V Ommen S R Late health status of patients undergoing myectomy for obstructive hypertrophic cardiomyopathy Ann Thorac Surg 2021 111 06 1867 1875 33127406 10.1016/j.athoracsur.2020.09.011

[5] SAM-Registry Study Investigators Cardiothoracic Surgery Registration Committee of the Netherlands Heeringa T JP Hegeman R MJJ Koop Y Surgical myectomy for hypertrophic cardiomyopathy: procedural volume and outcomes Eur Heart J 2025 ehaf560 10.1093/eurheartj/ehaf560 PMC13191829 40878834

[6] Alashi A Smedira N G Hodges K Outcomes in guideline-based class I indication versus earlier referral for surgical myectomy in hypertrophic obstructive cardiomyopathy J Am Heart Assoc 2021 10 01 e016210 33342243 10.1161/JAHA.120.016210 PMC7955478

[7] Vriesendorp P A Schinkel A FL Soliman O II Long-term benefit of myectomy and anterior mitral leaflet extension in obstructive hypertrophic cardiomyopathy Am J Cardiol 2015 115 05 670 675 25591899 10.1016/j.amjcard.2014.12.017

[8] Ommen S R Mital S Burke M A 2020 AHA/ACC guideline for the diagnosis and treatment of patients with hypertrophic cardiomyopathy: a report of the American College of Cardiology/American Heart Association Joint Committee on Clinical Practice Guidelines Circulation 2020 142 25 e558 e631 33215931 10.1161/CIR.0000000000000937

[9] Kofflard M J van Herwerden L A Waldstein D J Initial results of combined anterior mitral leaflet extension and myectomy in patients with obstructive hypertrophic cardiomyopathy J Am Coll Cardiol 1996 28 01 197 202 8752814 10.1016/0735-1097(96)00103-9

[10] Morrow A G Reitz B A Epstein S E Operative treatment in hypertrophic subaortic stenosis. Techniques, and the results of pre and postoperative assessments in 83 patients Circulation 1975 52 01 88 102 1169134 10.1161/01.cir.52.1.88

[11] Pinheiro J C Bates D M Mixed-Effects Models in S and S-PLUS New York Springer-Verlag 2000

[12] STS AATS EACTS Akins C W Miller D C Turina M I Guidelines for reporting mortality and morbidity after cardiac valve interventions Ann Thorac Surg 2008 85 04 1490 1495 18355567 10.1016/j.athoracsur.2007.12.082

[13] Hodges K Rivas C G Aguilera J Surgical management of left ventricular outflow tract obstruction in a specialized hypertrophic obstructive cardiomyopathy center J Thorac Cardiovasc Surg 2019 157 06 2289 2299 30782406 10.1016/j.jtcvs.2018.11.148

[14] Collis R Watkinson O O'Mahony C Long-term outcomes for different surgical strategies to treat left ventricular outflow tract obstruction in hypertrophic cardiomyopathy Eur J Heart Fail 2018 20 02 398 405 29148156 10.1002/ejhf.1038

[15] Desai M Y Bhonsale A Smedira N G Predictors of long-term outcomes in symptomatic hypertrophic obstructive cardiomyopathy patients undergoing surgical relief of left ventricular outflow tract obstruction Circulation 2013 128 03 209 216 23770748 10.1161/CIRCULATIONAHA.112.000849

[16] Hong J H Nguyen A Schaff H V Management of the mitral valve in patients with obstructive hypertrophic cardiomyopathy Indian J Thorac Cardiovasc Surg 2020 36 01 34 43 33061183 10.1007/s12055-019-00817-y PMC7525927

[17] Hong J H Schaff H V Nishimura R A Mitral regurgitation in patients with hypertrophic obstructive cardiomyopathy: implications for concomitant valve procedures J Am Coll Cardiol 2016 68 14 1497 1504 27687190 10.1016/j.jacc.2016.07.735

[18] Holst K A Hanson K T Ommen S R Nishimura R A Habermann E B Schaff H V Septal myectomy in hypertrophic cardiomyopathy: national outcomes of concomitant mitral surgery Mayo Clin Proc 2019 94 01 66 73 30611455 10.1016/j.mayocp.2018.07.022

[19] van der Lee C Kofflard M JM van Herwerden L A Vletter W B ten Cate F J Sustained improvement after combined anterior mitral leaflet extension and myectomy in hypertrophic obstructive cardiomyopathy Circulation 2003 108 17 2088 2092 14517170 10.1161/01.CIR.0000092912.57140.14

[20] Huurman R Schinkel A FL de Jong P L van Slegtenhorst M A Hirsch A Michels M Impact of sex on timing and clinical outcome of septal myectomy for obstructive hypertrophic cardiomyopathy Int J Cardiol 2021 323 133 139 32841616 10.1016/j.ijcard.2020.08.059

[21] Lentz Carvalho J Schaff H V Nishimura R A Is anterior mitral valve leaflet length important in outcome of septal myectomy for obstructive hypertrophic cardiomyopathy? J Thorac Cardiovasc Surg 2023 165 01 79 870 33632527 10.1016/j.jtcvs.2020.12.143

