# Supplementary material for: Surgical Myectomy with Anterior Mitral Leaflet Extension Versus Isolated Myectomy in Patients with Hypertrophic Obstructive Cardiomyopathy
Source: Thorac Cardiovasc Surg. 2026 Jan 7;74(6):468–76. doi: 10.1055/a-2768-2815 (PMC13521660; doi:10.1055/a-2768-2815)
Supplement: Supplementary file 1 [file 10-1055-a-2768-2815_29430298.pdf]

Data Supplementary Material.

Supplemental Methods

S1.1 Data collection and follow-up

Data uploaded to the national database of the Netherlands Heart Registration with the use of a predefined data collection form was used for this study. The Netherlands Heart Registration database is a prospective mandatory database that contains data on all cardiac surgical interventions performed in the Netherlands, including information on preoperative patient characteristics, intervention variables, and outcomes.19 Multiple quality checks are performed on the data during the data collection and cardiac centres are audited regularly.20 The basis for data collection was reported elsewhere.21 All data was manually reviewed, incorporating supplementation and rectification in instances of missing or invalid information and conforming to information in the electronic medical records. The authors requested preoperative and follow-up outpatient clinic reports if patients were referred from and to other hospitals. Follow-up transthoracic echocardiography images were requested if patients were referred to another hospital after surgery. The digitally stored transthoracic echocardiography images from four time points – preoperatively, postoperatively during hospital admission, at the first outpatient visit, and at three-year follow-up – were re-evaluated by two imaging cardiologists (M.G. and M.J.C.). The parameters were assessed according to the recommendation of the European Society of Cardiovascular Imaging and recommendations from the Society of Echocardiography.1,22,23 The methods used for parameter evaluation can be found in the Supplementary material, Data S1.

For the follow-up, all patients were contacted for a telephone consultation to evaluate the HCM family history, and heart failure symptoms and to assign a New York Heart Association (NYHA) functional score. For patients who were lost to follow-up for a telephone interview, the NYHA functional score was collected from the outpatient reports. This score was assigned as missing when the patient died or if no outpatient report was available.

S1.2 Echocardiographic measurements:

We assessed the parameters according to the recommendation of the European Society of Cardiovascular Imaging and recommendations from the Society of Echocardiography (EACVI) (1,2). The following echocardiogram measurements – interventricular septal thickness in diastole (IVSd), left atrial volume index (LAVI), left atrial dimension (LAD), tricuspid annular plane systolic excursion (TAPSE), and left ventricular ejection fraction (LVEF), provoked left ventricular outflow tract (LVOT) gradient, resting LVOT gradient,

The Thoracic and Cardiovascular Surgeon | DOI 10.1055/a-2768-2815 | © The Author(s).

systolic anterior motion (SAM), mitral regurgitation (MR) – were assessed at the four-time points.

IVSd

The maximum IVSd was measured at the end-diastole in the parasternal long-axis (PLAX) in millimeter (mm). In all postoperative echocardiograms, the measurement of IVSd was solely taken at the basal septal level where the myectomy was performed. Care was taken to include only the myocardium and not papillary muscles and right ventricular structures.

Left atrium

The LAD was assessed in the end-systolic phase, measured perpendicular to the long axis of the left atrium. The LAVI was assessed apical in the two- and four-chamber views.

Function

The LVEF was estimated by eyeballing in the 2-chamber and 4-chamber views. The TAPSE was measured in the apical four-chamber view during end-systole.

LVOT

The resting LVOT pressure gradient was estimated using continuous wave Doppler echocardiography, employing a simplified Bernoulli equation. If available, the provoked LVOT gradient, provoked by exercise or Valsalva, was estimated using continuous wave Doppler echocardiography. Particular attention was given to avoiding contamination of the LVOT waveform by the mitral regurgitation jet. The presence of valvular SAM was assessed in PLAX and four-chamber.

MR

The presence of MR was categorized into four grades: grade 1 (none), grade 2 (trace / mild), grade 3 (moderate), and grade 4 (severe).

S2 Model structure

All models included time and stratified surgical procedures (AMLE+SM and isolated surgical myectomy) to evaluate the progression of echocardiographic outcomes. As the progression of outcomes over time was different before and after surgery, the time effect was separated by incorporating an additional dummy variable separating pre- and post-surgery time effects. The difference between procedures was included with an interaction of the procedure with the effects of time: This was tested with a likelihood ratio test.(3)

The Thoracic and Cardiovascular Surgeon | DOI 10.1055/a-2768-2815 | © The Author(s).

Random effects for intercept and time were incorporated in all models. However, in the analyses of LVOT gradient, TAPSE, and LVEF, the random effect for time was excluded, as the corresponding variance component was close to zero. All analyses included an adjustment for age and sex as potential confounders.

Residual plots were used to assess the model's assumptions (normality and homoscedasticity). Normal distributed variables (LVEF and TAPSE) were plotted as means with 95% confidence intervals (95% CIs). Non-normally distributed variables (LVOT gradient, IVSd, LAVI, and LAD) underwent log transformation and were plotted as medians with 95% CIs.(3)

1. Lancellotti P, Pibarot P, Chambers J, La Canna G, Pepi M, Dulgheru R, et al. Multi-modality imaging assessment of native valvular regurgitation: an EACVI and ESC council of valvular heart disease position paper. Eur Heart J Cardiovasc Imaging. 2022 Apr 18;23(5):e171–232.

2. Lang RM, Badano LP, Mor-Avi V, Afilalo J, Armstrong A, Ernande L, et al. Recommendations for Cardiac Chamber Quantification by Echocardiography in Adults: An Update from the American Society of Echocardiography and the European Association of Cardiovascular Imaging. Journal of the American Society of Echocardiography. 2015 Jan;28(1):1-39.e14.

3. Fitzmaurice GM, Laird NM, Ware JH. Applied Longitudinal Analysis. Wiley; 2011.

Supplemental outcomes

Symptomatic relief

In the SM+AMLE group, 17 (53%) of the patients at their most recent follow-up were in NYHA functional Class I, 7 (22%) were in NYHA functional Class II, 4 (13%) were in NYHA functional Class III, and none of the patients were in NYHA functional Class IV. The NYHA functional class was unknown in 4 patients (13%) at the most recent follow-up. In the isolated surgical myectomy group, 15 of the patients at their most recent follow-up (54%) were in NYHA functional Class I, 9 (32%) were in NYHA functional Class II, and none were in NYHA functional Classes III or IV, at the most recent follow-up. NYHA functional Class was unknown in 1 patient (4%).

The Thoracic and Cardiovascular Surgeon | DOI 10.1055/a-2768-2815 | © The Author(s).

Supplemental Tables

Supplementary Table S1. Concomitant procedures of the excluded patients.

Concomitant procedure	Patients (n, [%])
CABG	
CABG	5 (9)
CABG + AVR	4 (8)
CABG + MV surgery	3 (6)
AVR	
AVR	13 (25)
AVR + MV surgery	7 (13)
AVR + aorta	2 (4)
AVR + MV + aorta	1 (2)
MV surgery	
MV surgery	11 (21)
MV surgery + PVR	2 (4)
MV surgery + TVR	3 (6)
TVR	1 (2)
Right ventricle ESM	1 (2)
**Total**	**53 (100)**

Abbreviations: AVR, aortic valve replacement; CABG, coronary artery bypass grafting; MV, mitral valve; PVR, pulmonary valve replacement; and TVR, tricuspid valve replacement.

The Thoracic and Cardiovascular Surgeon | DOI 10.1055/a-2768-2815 | © The Author(s).

Supplementary Table S2. Missing values of preoperative patient characteristics of all included surgical myectomy procedures.

	Total (n = 59)	SM+AMLE (n = 34)	Isolated surgical myectomy (n = 25)
**Age**	**0 (0%)**	0 (0%)	0 (0%)
**Gender**	**0 (0%)**	0 (0%)	0 (0%)
**BMI**	**0 (0%)**	0 (0%)	0 (0%)
**Creatinine (Renal insufficiency)**	**0 (0%)**	0 (0%)	0 (0%)
**Euroscore II**	**0 (0%)**	0 (0%)	0 (0%)
**Prior cardiac surgery**	**0 (0%)**	0 (0%)	0 (0%)
**Prior ASA**	**0 (0%)**	0 (0%)	0 (0%)
**Atrial fibrillation history**	**0 (0%)**	0 (0%)	0 (0%)
**ICD history**	**0 (0%)**	0 (0%)	0 (0%)
**Diabetes**	**0 (0%)**	0 (0%)	0 (0%)
**Pacemaker history**	**0 (0%)**	0 (0%)	0 (0%)
**LBBB**	**0 (0%)**	0 (0%)	0 (0%)
**RBBB**	**0 (0%)**	0 (0%)	0 (0%)
**Pathogenetic DNA variants**	**27 (46%)**	17 (50 %)	10 (40%)
**NYHA**	**0 (0%)**	0 (0%)	0 (0%)
**Concomitant surgery**	**0 (0%)**	0 (0%)	0 (0%)
**Systolic LVF**	**0 (0%)**	0 (0%)	0 (0%)
**LAVI**	**2 (3%)**	0 (0%)	0 (0%)
**LADi**	**2 (3%)**	2 (6%)	0 (0%)
**Resting LVOT gradient**	**0 (%)**	0 (0%)	0 (0%)
**Provocative LVOT gradient**	**19 (32%)**	15 (44%)	4 (16%)

The Thoracic and Cardiovascular Surgeon | DOI 10.1055/a-2768-2815 | © The Author(s).

**Mitral regurgitation**	**0 (0%)**	0 (0%)	0 (0%)
**Valvular SAM**	**0 (0%)**	0 (0%)	0 (0%)
**IVSD**	**0 (0%)**	0 (0%)	0 (0%)
**TAPSE**	**0 (0%)**	0 (0%)	0 (0%)
**ACE-inhibitors**	**0 (0%)**	0 (0%)	0 (0%)
**Anticoagulantia**	**0 (0%)**	0 (0%)	0 (0%)
**ARB**	**0 (0%)**	0 (0%)	0 (0%)
**B-blockers**	**0 (0%)**	0 (0%)	0 (0%)
**Calcium channel blockers**	**0 (0%)**	0 (0%)	0 (0%)
**Disopyramide**	**0 (0%)**	0 (0%)	0 (0%)
**Diuretics**	**0 (0%)**	0 (0%)	0 (0%)

Abbreviations: ACE, angiotensin-converting enzyme; ARB, angiotensin II receptor blocker; ASA, alcohol septal ablation; BMI, body mass index; ICD, implantable cardioverter defibrillator; IVSd, interventricular septal thickness in diastole; IQR, interquartile range; LADi, left atrial dimension indexed; LAVI, left atrial volume index; LBBB, left bundle branch block; LVOT, left ventricular outflow tract; MR, mitral regurgitation; RBBB, right bundle branch block; SAM, systolic anterior motion; SM, surgical myectomy; SM+AMLE, surgical myectomy with anterior mitral valve leaflet extension; TAPSE, tricuspid annular plane systolic excursion.

The Thoracic and Cardiovascular Surgeon | DOI 10.1055/a-2768-2815 | © The Author(s).

Supplementary Table S3. Missing values of intra-operative, and postoperative variables of all included surgical myectomy procedures.

	Total (n = 59)	SM+AMLE (n = 34)	Isolated surgical myectomy (n = 25)
**VSD**	**0 (0%)**	0 (0%)	0 (0%)
**ACC time in minutes**	**0 (0%)**	0 (0%)	0 (0%)
**CPB time in minutes**	**0 (0%)**	0 (0%)	0 (0%)
**Surgical myectomy**	**0 (0%)**	0 (0%)	0 (0%)
**Mortality**	**0 (0%)**	0 (0%)	0 (0%)
**New pacemaker**	**0 (0%)**	0 (0%)	0 (0%)
**New ICD**	**0 (0%)**	0 (0%)	0 (0%)
**Stroke**	**0 (0%)**	0 (0%)	0 (0%)
**Heart rhytm complications**	**0 (0%)**	0 (0%)	0 (0%)
**LBBB postoperative**	**0 (0%)**	0 (0%)	0 (0%)
**Surgical reoperation**	**0 (0%)**	0 (0%)	0 (0%)
**Mediastinitis**	**0 (0%)**	0 (0%)	0 (0%)
**Bleeding**	**0 (0%)**	0 (0%)	0 (0%)
**Systolic LVF**	**0 (4%)**	0 (2%)	0 (0%)
**LAVI**	**2 (3%)**	2 (3%)	1 (4%)
**LADI**	**1 (2%)**	1 (3%)	0 (0%)
**Resting LVOT gradient**	**0 (0%)**	0 (0%)	0 (0%)
**Provocative LVOT gradient**	**20 (33%)**	11 (32%)	9 (36%)
**Mitral regurgitation**	**0 (0%)**	0 (0%)	0 (0%)
**Valvular SAM**	**0 (0%)**	0 (0%)	0 (0%)
**IVSd**	**0 (0%)**	0 (0%)	0 (0%)

The Thoracic and Cardiovascular Surgeon | DOI 10.1055/a-2768-2815 | © The Author(s).

**TAPSE**	**0 (0%)**	0 (0%)	0 (0%)

Abbreviations: ACC, aortic cross clamp; CPB, cardiopulmonary bypass; ICD, implantable cardioverter defibrillator; IVSD, inter ventricular septal thickness in diastole; LADI, left atrial dimension index; LAVI, left atrial volume index; LBBB, left bundle branch block; LVF, left ventricular function; SAM, systolic anterior motion; TAPSE, tricuspid annular plane systolic excursion; VSD, ventricular septal defect.

The Thoracic and Cardiovascular Surgeon | DOI 10.1055/a-2768-2815 | © The Author(s).

Table S4. Postoperative echocardiographic parameters.

	Postoperative			*First FU*			*3-year FU*		
	SM+AMLE (n = 34)	*Isolated SM (n = 25)*	*p* value	*SM+AMLE (n=34)*	*Isolated SM (n = 25)*	*p* value	*SM+AMLE (n = 34)*	*Isolated SM (n = 25)*	*p* value
Follow-up in months (mean, [SD])	*0 (0)*	*0 (0)*	*0.33*	*5 (3)*	*5 (3)*	*0.25*	*36 (5)*	*36 (7)*	*0.48*
Systolic LVF in % (median, [IQR])			*0.90*			*0.14*			*0.91*
Good (EF ≥ 50%)	*33 (97)*	*24 (96)*		*28 (82)**	*20 (80)**		*28 (82)**	*20 (80)**	
Impaired (EF 40%–50%)	*1 (3)*	*1 (4)*		*1 (3)*	*1 (4)*		*1 (3)*	*1 (4)*	
Moderately reduced (EF 30%–40%)	*0*	*0*		*0*	*0*		*0*	*0*	
LAVI in cm^2 (mean, [SD])	*55 (16)*	*52 (13)*	*0.54*	*42 (12)*	*35 (11)*	*0.01*	*47 (13)*	*38 (9)*	*0.01*
LADi in mm (mean [SD])	*21 (3)*	*21 (3)*	*0.60*	*21 (4)*	*20 (4)*	*0.68*	*21 (4)*	*21 (3)*	*0.62*
SAM (n, (%))	*0*	*2 (8)*	*0.18*	*1 (4)*	*0*	*NA*	*1 (4)*	*1 (5)*	*NA*
Resting LVOT gradient in mmHg (median, [range])	*9 (2–27)*	*8 [4–32]*	*0.90*	*5 [2–16]*	*8 [3–12]*	*0.13*	*7 [2–20]*	*6 [3–13]*	*0.84*
Provocative LVOT gradient in mmHg (median, [range])	*14 [5–42]**	*10 [5–60]**	*0.11*	*26 [4–60]**	*22 [15–25]**	*0.81*	*9 [4–17]**	*14 [6–21]**	*0.67*
IVSd (mean, [SD])	*11 (2)*	*11 (2)*	*0.26*	*12 (2)*	*11 (2)*	*0.03*	*12 (2)*	*11 (2)*	*0.27*
Mitral regurgitation (n, [%])			*0.57*			*NA*			*NA*
MR grade 1+2	*33 (97)*	*23 (92)*		*29 (82)*	*21 (84)*		*27 (73)**	*20 (80)**	
MR grade 3+4	*1 (3)*	*2 (8)*		*0*	*0*		*2 (6)*	*1 (4)*	
TAPSE (mean, [SD])	*17 (3)*	*17 (3)*	*0.54*	*19 (3)*	*18 (3)*	*0.28*	*18 (2)*	*18 (3)*	*0.91*

Abbreviations: FU. Follow-up; IVSd, interventricular septal thickness in diastole; LADi, left atrial dimension indexed; LAVI, left atrial volume index; LVF, left ventricular fraction; LVOT, left ventricular outflow tract; MR, mitral regurgitation; SAM, systolic

anterior motion; SM, surgical myectomy; SM+AMLE, surgical myectomy with anterior mitral valve leaflet extension; TAPSE, tricuspid annular plane systolic excursion. *Missing values ≥ 15%.

Supplemental Figures

Supplementary Figure 1. Visual echocardiographic measurements for (A) SM+AMLE (n = 34) and (B) isolated surgical myectomy (n=25). SAM and mitral regurgitation measurements at four time points: (1) preoperatively, (2) postoperatively during

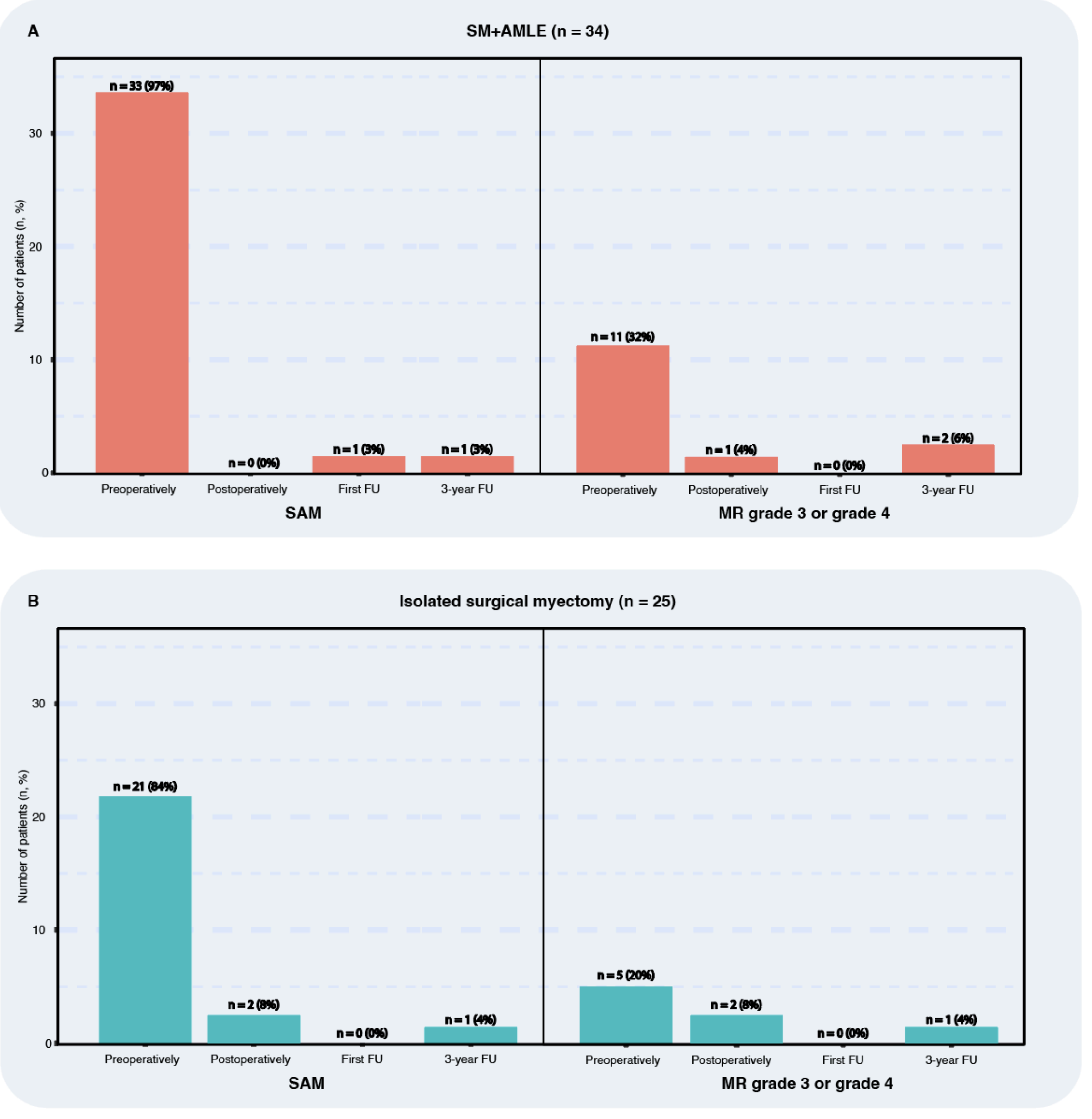

hospital admission, (3) at first out-patient follow-up, (4) at a three-year follow-up.

Abbreviations: FU, follow-up, MR, mitral regurgitation; SAM, systolic anterior motion; SM+AMLE, surgical myectomy with anterior mitral valve leaflet extension.
